# Electroacupuncture Could Influence the Expression of IL-1*β* and NLRP3 Inflammasome in Hippocampus of Alzheimer's Disease Animal Model

**DOI:** 10.1155/2018/8296824

**Published:** 2018-07-12

**Authors:** Jing Jiang, Ning Ding, Kang Wang, Zhigang Li

**Affiliations:** ^1^Beijing University of Chinese Medicine, Beijing 100029, China; ^2^Dongfang Hospital, Beijing University of Chinese Medicine, Beijing 100078, China

## Abstract

**Background:**

Effective therapies for Alzheimer's disease (AD) are still being explored. Electroacupuncture with traditional Chinese medicine theory may improve spatial learning and memory abilities and glucose metabolism rates in an animal model of AD. However, the mechanism of electroacupuncture in intervention of AD is still unclear. According to recent studies of AD mechanisms, the NLRP3 inflammasome regulated the expression of IL-1*β* in the brain which may mediate AD related processes. Therefore, in our study, we intend to explore the possible relation between electroacupuncture and the expression of NLRP 3 inflammasome in the hippocampus of an AD animal model.

**Method:**

In this study, 7.5-month-old male senescence-accelerated mouse prone 8 (SAMP8) mice were used as an AD animal model, which were randomly divided into two groups: Alzheimer's disease model group (AD group) and electroacupuncture group (EA group). In the control paradigm, 7.5-month-old male SAMR1 mice were used as the normal control group (N group). DU20, DU26, and EX-HN3 were selected as the acupuncture points, and after a 15-day treatment of electroacupuncture, we used immunohistochemistry and Western blotting to examine the expression of IL-1*β* and NLRP3, ASC, and Caspase-1 in the hippocampus of the AD animal model.

**Results:**

Compared with N group, IL-1*β*, NLRP3, ASC, and Caspase-1 positive cells in AD group were increased, and the relative expression of all above proteins significantly increased (P < 0.01). Compared with AD group, the expression of IL-1*β*, NLRP3, ASC, and Caspase-1 in EA group was significantly decreased (P < 0.01).

**Conclusion:**

Electroacupuncture treatment could inhibit the inflammation reaction in the hippocampus of SAMP8 mice. What is more, the possible mechanism of electroacupuncture reduced the expression of IL-1*β* and NLRP3 inflammasome relative protein.

## 1. Introduction

Alzheimer's disease (AD), as the most common type of dementia, represents about 60%-80% of these diseases [1]. “World Alzheimer's Disease Report” [2] reported that there were currently 47 million people worldwide suffering from Alzheimer's disease, with a diagnosis every 3.2 seconds. With the aging global population aging, the number of AD patients will be greater than 131 million by 2050. In addition, recent studies indicate that the prevalence of AD in developing countries is particularly high [3]: in 2015, 58% of AD patients were from developing countries, but this figure is expected to increase in 2030 and 2050 to 63% and 68%, respectively. As a result, AD has become one of the major challenges facing the world (especially in developing countries) in the public health sector.

A large number of studies have concluded that there is no currently effective treatment to terminate or reverse AD. Therefore, the goal of clinical treatment of AD is to prevent the occurrence of AD, delay the disease process, and/or stabilize or improve its symptoms [4]. In traditional Chinese medicine theory, acupuncture therapy based on meridian and acupoints plays an active role in the clinical treatment of AD [5]. We have found that electroacupuncture could improve the spatial learning and memory ability and glucose metabolism rate level in an AD animal model [6, 7]. We have also reported that electroacupuncture could alleviate the neuroinflammation reaction in brain of ischemic stroke [8], post-stroke cognitive impairments [9], and cognitive deficits of CCI rats [10]. However, the mechanism of electroacupuncture is still being explored for expanding the use of this therapy in real clinical situations.

Researchers are actively investigating the mechanisms of AD, and the accumulation of insoluble amyloid-*β*, hyperphosphorylation of Tau protein, and neuroinflammation caused by the innate immune system in the brain have gained greater attention [11]. Recent data emerging from genetic studies, clinical imaging, and animal experimentation point to an intimate interaction of innate immune system in AD processes [12]. Researchers have reported that, in NOD-like receptor (NLR) family, the pyrin domain containing 3 (NLRP3) inflammasome, which regulates the expression of IL-1*β* in brain, is active in neurodegenerative disease, especially in AD [13]. Amyloid *β* in brain, as one of the triggers of NLRP3 inflammasome, can induce the activation of the NLRP3 inflammasome and induce overexpression of IL-1*β* and neuroinflammation, ultimately accelerating AD [14]. Therefore, inhibiting activation of the NLRP3 inflammasome and expression of IL-1*β* is regarded as one possible therapy for AD [15].

What is the mechanism of electroacupuncture in intervention on AD? Could electroacupuncture inhibit the activation of NLRP3 inflammasome and expression of IL-1*β* in AD animal model? To answer these questions, we designed the present study. 7.5-month-old male senescence-accelerated mouse prone 8 (SAMP8) mice were used as an AD animal model, and immunohistochemistry and Western blot were used to examine the expression of IL-1*β*, NLRP3, ASC, and Caspase-1 proteins in hippocampus of the brain.

## 2. Materials and Method

### 2.1. Animals

Senescence-accelerated mouse prone 8 (SAMP8) mice and cognate normal senescence-accelerated mouse-R1 (SAMR1) mouse breeding pairs were kindly provided by Professor Takeda at Kyoto University, Japan. All animals were male and specific pathogen free (SPF), weighing 30±2 g. They were housed in a barrier facility at the Experimental Animal Centre of First Teaching Hospital of Beijing University of Traditional Chinese Medicine, under controlled temperature (24 ± 2°C) and 12h/12h dark-light cycle, with sterile drinking water and standard pellet diet* ad libitum*. All experiments were performed according to the National Institute of Health Guide for the Care and Use of Laboratory Animals (NIH publications number 80-23). Twenty 7.5-month-old male SAMP8 mice were divided into two groups (n = 10 per group), including SAMP8 Alzheimer's disease control (AD group) and electroacupuncture (EA group). Ten 7.5-month-old male SAMR1 mice composed the normal control (N) group.

### 2.2. Electroacupuncture Manipulation

In the EA group, electroacupuncture treatment was performed 20 minutes per day, one time a day for 15 days (no treatment on 8th day). Acupuncture points included DU20* Baihui*, DU 26* Shuigou*, and EX-HN3* Yintang* (significant extra points); the locations of these points were according to the National Acupuncture Society for Experimental Research developed by the “laboratory animal acupuncture atlas”. 0.5-inch needles (*Huatuo, Beijing, China*) were used for treatment. The pricking method was used for DU 26* Shuigou*, and the flat thorn method for DU20* Baihui* and EX-HN3* Yintan*g (needle depth was 0.5 cm). The needles were taped and connected to the HANS- LH202 electroacupuncture device (*Peking University Institute of Science Nerve and Beijing Hua Wei Industrial Development Company, Beijing, China*), with sparse wave at 2 Hz, 2 V, and 0.6 mA.

In the N and AD groups, no treatment was carried out other than handling and fixing once daily for 15 days, except on the 8th day, to ensure the same treatment conditions.

### 2.3. Immunohistochemistry

After the treatment, 4 mice were chosen from each group for immunohistochemical examination. They were anesthetized by intraperitoneal injection of 10% chloral hydrate at 0.35 mL/100 g body weight. Three minutes later, the chest was opened and the heart was exposed; intubation was performed from the left ventricle to the ascending aorta with quick injection of 100 ml saline. Then, the right atrial appendage was cut, and 4% paraformaldehyde was injected until the liver turned white with clear fluid flowing out from the right atrial appendage. After the perfusion, the mouse was decapitated and the whole brain extracted and placed on ice. Brains were then placed into 4% paraformaldehyde for paraffin embedding.

For immunohistochemistry, paraffin embedded brain tissue sections were deparaffinized with xylene and hydrated with graded alcohol. Then, the sections were treated with citric acid antigen repair buffer and washed with PBS (pH 7.4) three times with shaking, 5 minutes apart. After incubation with 3% hydrogen peroxide for 20 min in the dark to quench endogenous peroxidase, the sections were separately incubated with anti-A*β*1-42 antibody (1:50, ab10148), anti-NLRP3 antibody (1:500, orb76179), anti-ASC antibody (1:900, NBP1-68187), anti-Caspase-1 antibody (1:600, ab108362), and anti-IL-1*β* antibody (1:50, ab9787) overnight. Then, secondary antibodies were added for 30 min at room temperature, and detection was performed with DAB. Finally, the sections were dehydrated with graded alcohol and mounted. Micrographs of brain tissue samples were obtained at 400x magnification, and integral optical density (IOD) values were calculated using Image-Pro Plus 6.0 software.

### 2.4. Western Blotting

At the end of the treatment, the remaining 6 mice of each group were sacrificed by caudal dislocation. After decapitation, the whole hippocampus was dissected on a sterile working table. This procedure was performed on ice. The removed hippocampal tissue was placed in a cryopreserved tube, numbered, and stored in liquid nitrogen. After the operation, the hippocampus tissue was transferred to a refrigerator at – 80°C.

Total protein was extracted using cell lysate. Protein quantification was performed using the Bradford method. A 40 *μ*g sample of protein was added to sodium dodecyl sulfate polyacrylamide gel electrophoresis and transferred to polyvinylidene fluoride membranes. Incubation of primary antibodies was performed overnight at 4°C. Antibody dilution was as follows: NLRP3 (1:300,), ASC (1:500,), Caspase-1 (1:1000,), and IL-1*β* (1: 2,500,). This was followed by incubation with HRP-coupled anti-mouse or rabbit IgG antibody (1:5,000,) at 37°C for 1 hours. Target proteins on polyvinylidene fluoride membrane were visualized using ECL kit and captured using a BIO-RAD BioImaging System.

### 2.5. Statistical Analysis

All data are presented as mean ± SD for each group. One-way ANOVA was used with Western blot data. LSD tests were used to compare group pairs. Statistical significance was set at p<0.05. All statistical analyses were performed with the SPSS software V.17.0 (SPSS, USA).

## 3. Results

### 3.1. Expression of IL-1*β* and NLRP3 Related Proteins in Hippocampus: Seeing from Immunohistochemistry

**Figure 1:**
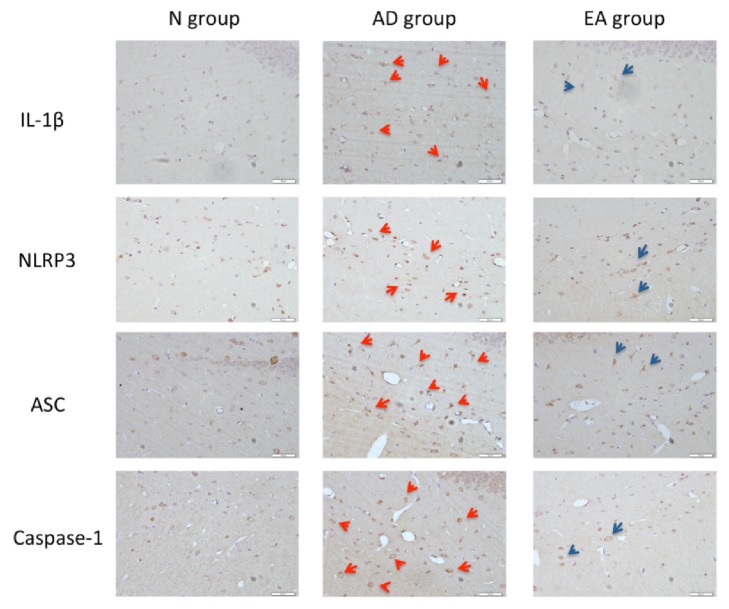
Expression of IL-1*β* and NLRP3 related proteins.

In the immunohistochemical results (Figure 1), the number of positive cells expressing IL-1*β* protein in hippocampus of mice in N group was low, and occasional positive cells were also lighter in staining. Compared with N group, the number of IL-1*β* protein positive cells in the same field of view of the hippocampus of the AD group was significantly higher (highlighted by red arrows). Compared with AD group, the number of IL-1*β* positive cells in the same field of view of the EA group was reduced and the staining was lighter (highlighted by blue arrows).

In addition, the expression of NLRP3 and related proteins (ASC and Caspase-1) was also increased in the AD group (highlighted by red arrows). Interestingly, these proteins were associated with the expression of IL-1*β*. We could still visually distinguish that the expression of NLRP3 and related protein (ASC and Caspase-1) positive cells in the EA group was decreased compared with the AD group, with lighter staining (highlighted by blue arrows).

### 3.2. Expression of IL-1*β* and NLRP3 Related Proteins in Hippocampus from Western Blots

**Figure 2:**
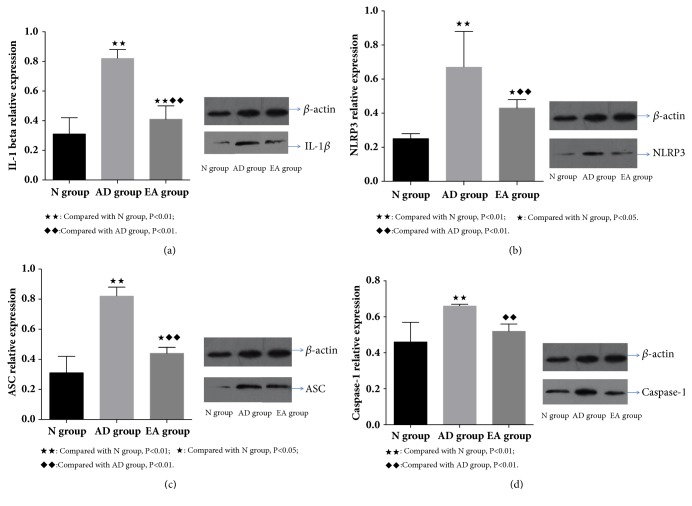


In the Western blot results (Figure 2), the relative expression of IL-1*β* and NLRP3 related proteins were obtained by comparing the gray value of each sample with *β*-actin, which was set as the internal reference.

Compared with N group, the expression of IL-1*β* protein in the hippocampus of the other two groups was significantly higher than that of N group (AD group: P < 0.01; EA group: P < 0.01). Compared with AD group, the expression of IL-1*β* protein in the hippocampus of the EA group was significantly decreased (P < 0.01, Figure 2(a)).

Compared with N group, the relative expression of NLRP3 protein in the hippocampus of other two groups was significantly higher (AD group: P < 0.01; EA group: P < 0.05). Compared with AD group, the expression of NLRP3 protein in the hippocampus of the EA group was significantly decreased (P < 0.01, Figure 2(b)).

Compared with N group, the relative expression of ASC protein in the hippocampus of other two groups was significantly higher (AD group: P < 0.01; EA group: P < 0.05). Compared with AD group, the expression of ASC protein in the hippocampus of the EA group was significantly decreased (P < 0.01, Figure 2(c)).

Compared with N group, the relative expression of Caspase-1 protein in hippocampus of AD group was significantly higher (P < 0.01). Although the EA group was higher than N group, this was not statistically significant (P = 0.212). However, compared with AD group, the relative expression of Caspase-1 protein in the hippocampus of the EA group was significantly decreased (P < 0.01, Figure 2(d)).

## 4. Discussion

### 4.1. Neuroinflammatory Response with Alzheimer's Disease

Several pathological and molecular biology studies of AD have indicated that the brain inflammatory response was central to the pathological changes of AD [16]. This kind of inflammatory response is different from the classical symptoms of acute inflammation (swelling, discoloration, and pain); it is a chronic inflammatory response. The cells involved in this inflammatory response are astrocytes and microglia which can produce a variety of cytokines, such as interleukin 1*α* (IL-1*α*), interleukin 1*β* (IL-1*β*), interleukin 6 (IL-6) and tumor necrosis factor (TNF), interleukin 8 (IL-8), macrophage inflammatory protein 1*α*, and monocyte chemo attractant protein 1, resulting in chronic inflammation, neuronal necrosis, and apoptosis, and eventually leading to impairment of cognitive function [17].

A large number of activated microglia and specific reactive astrocytes often accumulate around SPs [18]. Amyloid-*β* can have direct toxic effect on nerve cells and can also activate central nervous system microglia to release inflammatory mediators which trigger nerve cell injury signaling pathways, leading to neuronal damage [19]. Following activation of microglia cells, inflammatory factors can induce increases in amyloid-*β* precursor protein (APP) metabolism to increase amyloid-*β*, thereby increasing the inflammatory response reciprocally and accelerating the disease process [20]. Associated clinical studies have reported that long-term use of nonsteroidal anti-inflammatory drugs could reduce the incidence of Alzheimer's disease or delay its course [21]. Therefore, inhibition of the brain's neuroinflammatory response has become a novel target for treating Alzheimer's disease.

Previous work has concluded that the inflammatory mediator IL-1*β* is significantly increased in age-related brain diseases such as Alzheimer [22]. Our studies report a decreased expression of IL-1*β* following electroacupuncture in the hippocampus of the SAMP8 mouse brains. This suggests that electroacupuncture treatment could decrease central neuroinflammation via inhibition of IL-1*β* expression.

### 4.2. NLRP3 Inflammasome and Alzheimer's Disease

In the neurodegenerative progression of Alzheimer's disease, the deposition and aggregation of amyloid-*β* can activate a variety of receptors on the surface of microglia, stimulating the releasing of inflammatory cytokines such as IL-1*β* [23]. Recent studies suggest that NLRP3 inflammasome in microglia could be a new target for the treatment of AD [24].

NLRP3 is a macromolecular protein complex with a molecular weight of about 700 kD that exerts an exogenous microbial or endogenous risk sensor in the cytoplasm [25]. It is a molecular platform for the activation of caspase-1, which regulates the maturation and secretion of inflammatory cytokines such as IL-1*β*, IL-18, and IL-33 [26, 27]. In recent years, studies have shown that endogenous risk signals for the activation of NALP3 inflammatory include ATP, uric acid, reactive oxygen species (ROS), A*β* [28], extracellular matrix components, and lysosomes. Therefore, it is likely that the NLRP3 inflammasome plays a key role in type II diabetes [29], Alzheimer's disease [30], and other noninfectious inflammatory diseases [31].

The NLRP3 inflammasome consists of a NLRP3 scaffold and three ASC and Caspase-1 precursor components which are involved in the regulation of IL-1*β* activation and secretion [32]. Amyloid-*β* stimulation of NALP3 inflammatory agents induces IL-1*β* maturation and release, causing neuroinflammation which may be involved in AD etiology [33–35].

Apoptosis-associated speck-like protein containing a CARD (ASC), an important class of cell adapter protein, is involved in the composition of the NLRP3 inflammasome. ASC has a two-domain structure, the oligomeric domain (PYD), and the effect domain (CARD). Through these two domains, NLRP3 and Caspase-1 precursors can be recruited to form the NLRP3 inflammasome [36]. Immunohistochemistry studies indicate that the expression of ASC in microglia is positively correlated with the severity of inflammation [37].

Caspase-1 can initiate and perform a series of cellular procedures that lead to inflammatory responses or apoptosis [38]. After the assembly of NLRP3 inflammasome, Caspase-1 precursor is used to catalyze the formation of active Caspase-1. Caspase-1, also known as IL-1*β* converting enzyme (ICE) [39], is an effector of inflammatory cells, mediating the transition of inactive IL-1*β* precursor into mature IL-1*β* [40].

### 4.3. The Possible Mechanism of Electroacupuncture in Alzheimer's Disease

The actions of the NLRP3 inflammasome and its regulation of IL-1*β* expression have been explored in animal model neural studies [35]. Furthermore, clinical studies report upregulation of mRNA for NLRP3 inflammasome and its downstream effector IL-1*β* in both mild and severe AD patients [14]. Therefore, our study focused on the NLRP3 inflammasome to investigate the mechanisms of electroacupuncture in the treatment of AD.

In this study, we qualitatively analyzed the expression of IL-1*β*, NLRP3, ASC, and Caspase-1 by immunohistochemistry. We report positive expression of the above proteins in the hippocampus of 7.5-month-old SAMP8 mice (AD group), with the expression of positive cells (microglia) in the cytoplasm. Combined with Western blot results, the relative expression of the proteins in the AD group was significantly higher than in the N group, indicating that NLRP3 inflammasome-mediated neuroinflammation was present in the hippocampus of the AD model animals.

After the 15 days of electroacupuncture treatment, the spatial learning and memory abilities and the glucose metabolism rates are improved in the AD model animal [41]. In addition, from the present immunohistochemistry and Western blot results, we also documented that the expression of the inflammatory mediator IL-1*β* in the EA group was significantly lower than the levels in the AD group (P <0.01). We postulated that electroacupuncture may inhibit inflammation in the hippocampus of the brain. Interestingly, the relative expression of NLRP3, ASC, and Caspase-1 in the EA group was also significantly decreased compared with the AD group (P < 0.01). These data confirm our hypothesis that electroacupuncture can inhibit the neuroinflammatory response by modulating the NLRP3 inflammasome.

## 5. Conclusions

We report that electroacupuncture treatment can attenuate neuroinflammation in the hippocampus via inhibiting the expression of IL-1*β*, NLRP3, ASC, and Caspase-1. However, future genetic-level testing is needed to confirm and fully describe the beneficial effects of electroacupuncture.

## Data Availability

All the data (tables and figures) used to support the findings of this study are included within the article.
